# Importance of Polarizable Embedding for Absorption Spectrum Calculations of *Arabidopsis thaliana* Cryptochrome 1

**DOI:** 10.1021/acs.jpcb.4c02168

**Published:** 2024-06-24

**Authors:** Anders Frederiksen, Luca Gerhards, Peter Reinholdt, Jacob Kongsted, Ilia A. Solov’yov

**Affiliations:** † Institute of Physics, Carl von Ossietzky Universität Oldenburg, Carl-von-Ossietzky-Street 9-11, 26129 Oldenburg, Germany; ‡ Department of Physics, Chemistry, and Pharmacy, University of Southern Denmark, DK-5230 Odense M, Denmark; § Research Centre for Neurosensory Sciences, Carl von Ossietzky University of Oldenburg, Carl-von-Ossietzky Straße 9-11, 26111 Oldenburg, Germany; ∥ Center for Nanoscale Dynamics (CENAD), Carl von Ossietzky University of Oldenburg, Ammerländer Heerstr. 114-118, 26129 Oldenburg, Germany

## Abstract

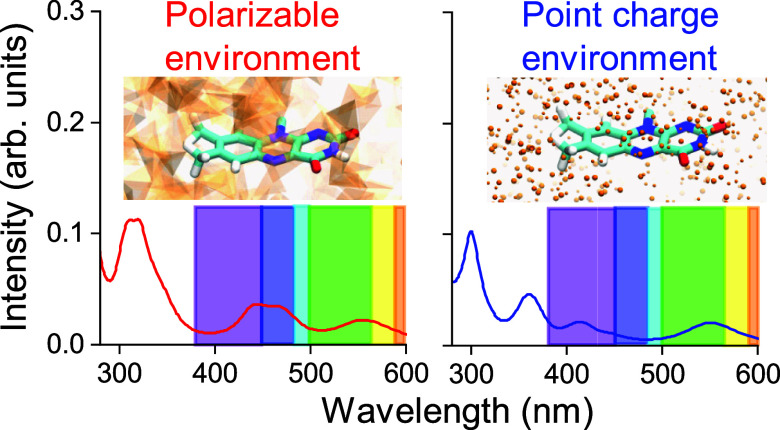

Cryptochromes are essential flavoproteins for circadian rhythms and avian magnetoreception. Flavin adenine dinucleotide (FAD), a chromophore within cryptochromes, absorbs blue light, initiating electron transfer processes that lead to a biological signaling cascade. A key step in this cascade is the formation of the FAD semiquinone radical (FADH^•^), characterized through a specific red-light absorption. The absorption spectra of FADH^•^ in cryptochromes are, however, significantly different from those recorded for the cofactor in solution, primarily due to protein-induced shifts in the absorption peaks. This study employs a multiscale approach, combining molecular dynamics (MD) simulations with quantum mechanical/molecular mechanical (QM/MM) methodologies, to investigate the influence of protein dynamics on embedded FADH^•^ absorption. We emphasize the role of the protein’s polarizable environment in the shaping of the absorption spectrum, crucial for accurate spectral predictions in cryptochromes. Our findings provide valuable insights into the absorption process, advancing our understanding of cryptochrome functioning.

## Introduction

The crucial role of light absorption in biological molecules is evident in processes such as photosynthesis, circadian rhythms, and avian magnetoreception.^1−6^ This function is usually delivered through light-absorbing chromophores embedded inside light-sensitive proteins, a notable example is the flavin adenine dinucleotide (FAD) cofactor, commonly found in proteins, whose biological functions often correlate with the redox state of FAD.^7^ The FAD cofactor plays an integral role in light-activated signal transduction across various species.^5,6,8,9^ Upon exposure to blue light, FAD often undergoes a series of redox transformations, where each state potentially serves a distinct function within its embedding protein.^8−10^

The light absorbing characteristics of FAD have been extensively documented in multiple proteins through computational simulations^11−16^ and empirical methods.^9,17−21^ Notably, transient absorption spectroscopy has elucidated the absorption attributes of FAD’s flavin moiety.^9,22^ However, comprehensive studies regarding other redox states of FAD remain rare.^11,12^ The semiquinone radical (FADH^•^) state is particularly intriguing due to its association with the prolonged signaling phase in specific proteins, like cryptochromes, which play a pivotal role in circadian rhythm modulation and magnetoreception.^8,23^

While in vitro analyses of cryptochromes provide insights into the general signaling dynamics,^9,18,23^ they rarely address the influence of the protein environment on electron excitation and transfer processes, the knowledge gap is gradually being filled by in silico research.^12,18,24,25^ Nevertheless, a significant challenge remains in accurately representing the protein environment theoretically, necessitating the integration of multiscale models.^26^ A notable example in this regard is the QM/MM models, which allow the simultaneous application of diverse theoretical frameworks to elucidate quantum properties within complex biological systems.^27−30^ In the QM/MM model approach, the designated core region of a molecular system is treated quantum mechanically, while the surrounding environment is approximated classically.^12,30−32^

**Figure 1:**
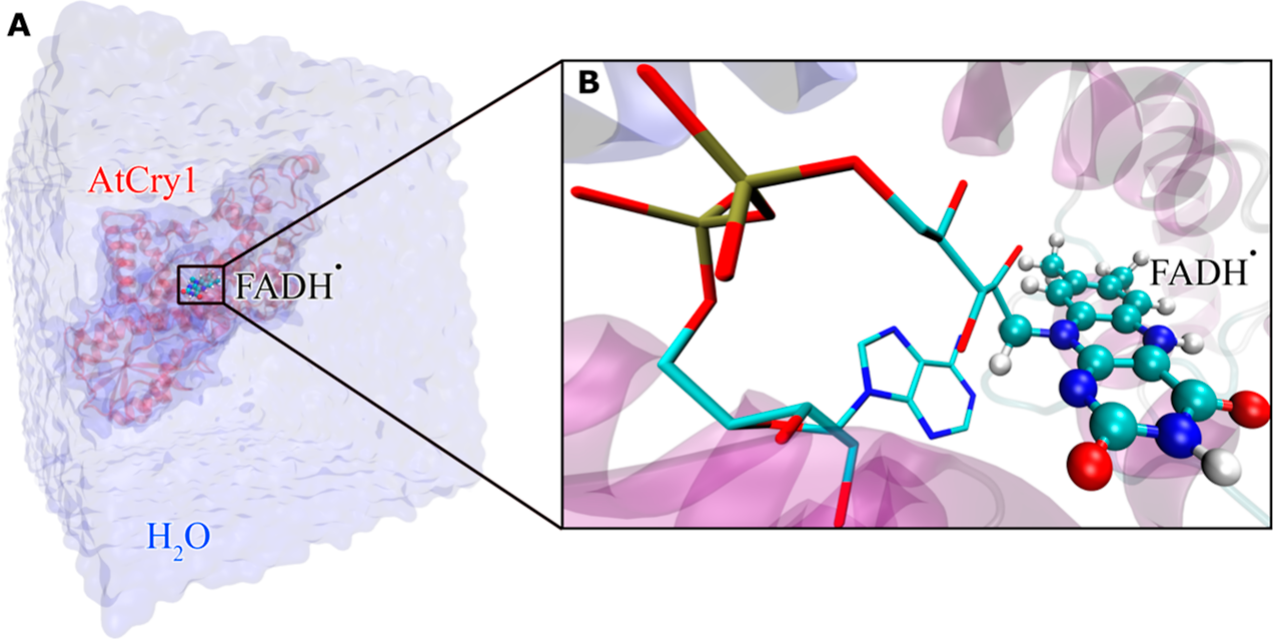
Model depiction of *Arabidopsis thaliana* cryptochrome 1 (AtCry1). (A) AtCry1 protein with the FADH^•^ cofactor immersed in a water box. The different representations indicate regions used in the calculations: solvent and protein (environment region) and flavin segment of FADH^•^ (quantum region). (B) AtCry1 with the quantum region emphasizing the flavin segment of FADH^•^, with the remaining part of FADH^•^ and the protein addressed using the PE method.

*Arabidopsis thaliana* cryptochrome 1 (AtCry1, Figure 1) which was studied extensively previously,^9,24,33,34^ serves as a great exemplary system for exploring how protein structures modulate light absorption of flavin.^35^ Given the thorough characterization of the protein over the years,^9,24,33,34^ AtCry1 provides a robust foundation for understanding FAD photophysics. However, a comprehensive description of the underlying processes still largely remains elusive, with the deficiency stemming from challenges in accurately representing the interactions between the chromophore and its surrounding molecular matrix. Historically, this environment has been modeled using classical molecular mechanical force fields, resulting in significant spectral shifts compared to experimental data.^36^

We conduct a quantum mechanical/molecular mechanical (QM/MM) investigation using the DALTON software package.^37^ The interaction of the chromophore, particularly the flavin segment of the FADH^•^ cofactor, with its environment is described using the polarizable embedding (PE) potential.^38−46^ Absorption properties are computed by treating the flavin cofactor as the core (quantum) region at the B3LYP theoretical level through the TD-DFT method. Additionally, we study flavin absorption inside AtCry1 by neglecting the polarizable environment’s impact on the FADH^•^ spectra through modeling the molecular environment of the chromophore in an electrostatic embedding; the approach is from here on referred to as electrostatic calculations.

## Methods

The atomistic structure of AtCry1 was obtained from the Protein Data Bank (PDB ID 1U3C),^35^ featuring a bound FAD cofactor. In our investigation, the FAD cofactor was transformed into its FADH^•^ form by adding an additional hydrogen atom to the N5 atom of the flavin moiety of FAD, as illustrated in Figure 1. The protein system was neutralized in a water box of 92.34 Å × 99.42 Å × 89.00 Å, with a concentration of 0.5 mM NaCl resulting in a system size of 83,404 atoms.

Molecular dynamics (MD) simulations were conducted using the CHARMM36 force field,^47,48^ with parametrizations specific for FADH^•^ derived previously.^3,12,25,49−51^

The NAMD program^52,53^ was employed to carry out the simulation. The structure underwent an initial conjugate gradient minimization consisting of 10,000 steps. Subsequently, the system was simulated with all atoms except the solvent atoms harmonically constrained for 1 ns. The spring constants of the harmonic restraints were set to 5 ps^–1^. This was followed by two equilibration stages: first, with the protein backbone harmonically restrained for 2 ns and then without any constraints for an additional 2 ns. The equilibration simulations used a 1 fs integration time step. The first two equilibration simulations were performed in the *NPT* ensemble, while the last equilibration was conducted in the *NVT* ensemble.

The MD simulations assumed a temperature of 310 K, regulated by the Langevin–Nosé–Hoover thermostat, and maintained at 1 bar pressure using a Langevin piston.^54,55^ Short-range interactions were managed using the 1–4 scaled exclusion approach,^56^ while long–range interactions were calculated with a cutoff distance of 12 Å and a switching distance of 10 Å. Long range electrostatic interactions were treated using the Particle Mesh Ewald (PME) summation method.^57^ The minimization and equilibration procedures were adapted from prior studies.^3,12,25,49−51,58,59^

The equilibrated system was subjected to a production run for 520 ns using a 2 fs integration time step, yielding 50 snapshots, with a 10 ns interval between each snapshot, ensuring statistical independence.

**Figure 2:**
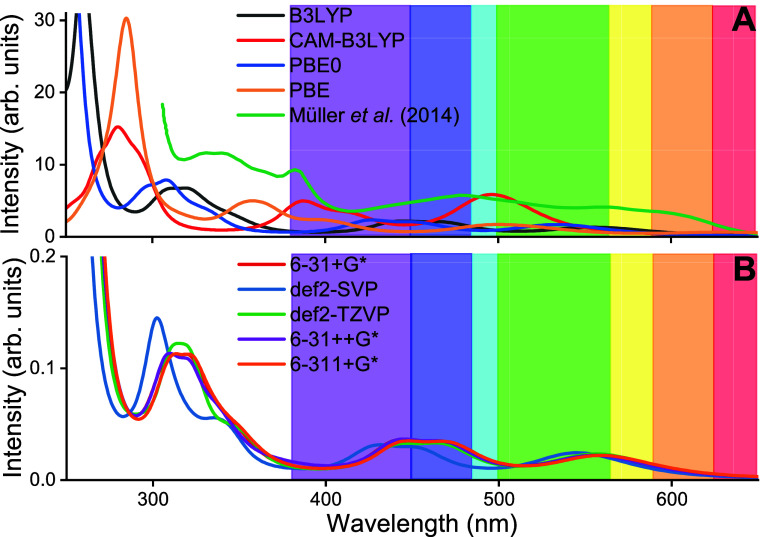
Methodological benchmarking of the different DFT functionals and basis sets used for the absorption spectra calculations of the flavin moiety of the FADH^•^ cofactor within solvated AtCry1. (A) Benchmarking of three common functionals against an earlier experimental study,^9^ all assuming the 6-31+G* basis set. (B) Benchmarking of the basis set, where both variants of the popular Pople basis set^65^ and *Alrichs* basis set^69^ were considered. All basis set calculations employed the B3LYP^64^ functional.

The flavin moiety of the FAD cofactor (the quantum region) was isolated, and point charges for the surrounding protein were assigned using the cost-effective polarizable protein potential (CP3)^60^ and solvent embedding potential (SEP).^61^ The point charge assignments were carried out using the PyFrame library,^62^ and the charges, dipoles and quadrupoles and polarizabilities of the adenosine dinucleotide moiety of the FAD cofactor were estimated using the Loprop method with the loprop-6-31+G* basis and PBE0, automatized in the PyFrame python package.^62^ The environment for the PE calculations thus consists of the solvent, the protein, and the adenosine dinucleotide moiety from the MD model, giving a total environment site number of 83,373. For investigating the effect of polarizable embedding (PE), two QM/MM model quantum chemistry (QC) calculations were performed. One set of calculations was based on the PE model, while the other set was in an electrostatic embedding and thus excluded the PE treatment. In both cases, the absorption spectra for each individual snapshot were computed using time-dependent density functional theory (TD-DFT)^63^ with the B3LYP method^64^ together with the 6-31+G* basis set.^65^ The B3LYP functional shows the best agreement with the experimental peak features compared to PBE0,^66^ PBE,^67^ and CAM-B3LYP^68^ (see Figure 2A). The 6-31+G* basis set,^65^ was also benchmarked against other basis sets in Figure 2B, and turned out to offer the best accuracy at a moderate computational cost. The TD-DFT calculations were conducted using the DALTON software package^37^ and based on these results (vertical excitation energies and oscillator strengths) the spectra were calculated using linear response theory. Afterward, broadening of the calculated spectra using an in-house Python script, with a Lorentzian line shape, L(ω), was calculated by
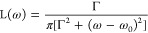
1Here, ω is the wavelength, ω_0_ is the center of each Lorentzian peak and Γ is the broadening factor, chosen to be 0.1 eV.

## Results and Discussion

The analysis presented in Figure 2B shows that for any studied basis set differing from def2-SVP there is a negligible difference in the computed spectra. The comparison of the results with the different functionals in Figure 2A, shows that PBE0 and B3LYP yield similar spectra, though B3LYP is slightly red-shifted compared to PBE0.

**Figure 3:**
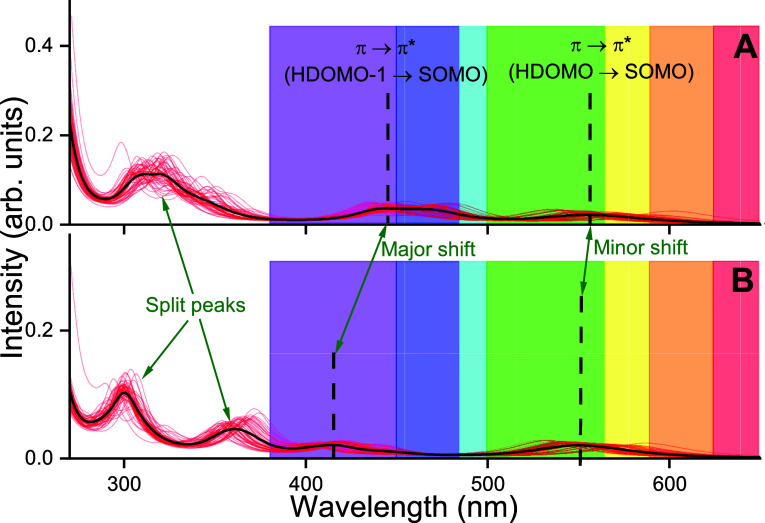
Averaged UV–vis absorption spectra (bold) of the flavin moiety of the FADH^•^ cofactor within solvated AtCry1 (see in Figure 1B) with each individual calculated spectrum shown as red lines. The spectra were computed using the B3LYP DFT functional with the 6-31+G* basis set. Highlighted with circles and arrows are the major differences between the two spectra, namely, minor and major shift of peak as well as a splitting of a peak in the UV. (A) Absorption spectrum accounting for the polarizable embedding of the environment of the flavin moiety, including polarizable water and protein with the VIS peaks highlighted and categorized (π or π*), stemming from a transition from the highest or second highest doubly occupied molecular orbital (HDOMO and HDOMO-1, respectively), to the singly occupied orbital (SOMO). (B) Photoexcitation spectrum of the flavin moiety inside AtCry1 using an electrostatic embedding.

The results of the CAM-B3LYP and PBE calculations have a much different spectral fingerprint and are not capturing the profile of the experimental spectrum. Due to the best alignment of B3LYP with the experimental results, further investigations are employed using only the B3LYP functional. Figure 3 illustrates the ultraviolet–visible (UV–vis) spectra of AtCry1, where the environment of the flavin moiety of the FADH^•^ cofactor is treated through the PE approach (see Figure 3A) and point-charge approximation (see Figure 3B).

Notably, the PE potential induces a red-shift in the absorption peak compared to that in the electrostatic model. This can be observed in the peak stemming from the transition of the highest doubly occupied molecular orbital (HDOMO) to the singly occupied molecular orbital (SOMO) at 555 nm for PE which is shifted by 5 to 550 nm. When compared to another study^34^ that utilized the multiconfigurational Complete Active Space Self-Consistent Field (CASSCF) method, this research reveals a greater discrepancy between the simulated and experimental first transition peaks. Specifically, the earlier study^34^ estimated the first peak at 495 nm, which differs significantly from the experimental value of 596 nm, while our findings are closer to the experiment with a value of 560 nm. Moreover, the second VIS peak (HDOMO-1 to SOMO transition Figure 3) at 444 nm is shifted by 30 to 414 nm for the electrostatic method compared to the PE method. This indicates that certain electronic excitations in proteins are more influenced by the polarizabilities of the protein electrostatic than other electronic excitations. The PE also shows a double peak around 320 nm, whereas the electrostatic spectrum has two distinct peaks. The doubling of the peaks indicates that the polarizable charges of the protein environment in the PE calculations are influencing the flavin moiety more than the electrostatic charges of the electrostatic model.

**Figure 4:**
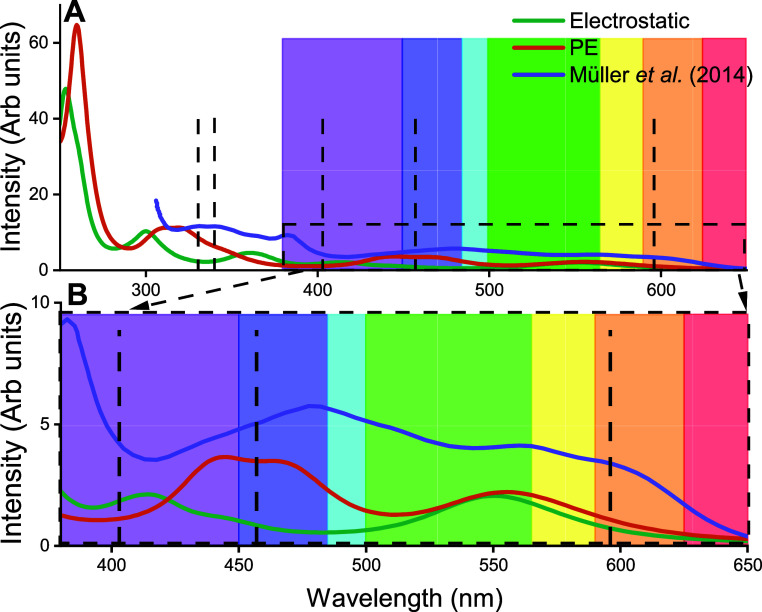
Comparison between the experimental absorption spectrum of AtCry1, the averaged absorption spectrum with account for the polarizable environment, of the flavin moiety (PE), with an electrostatic environment and an earlier experimental study. (A) UV–vis spectrum segment with peaks reported from earlier studies.^9,12^ (B) Zoomed view of the visible spectrum segment with three reported experimental excitations.^9^

Figure 4 shows a comparison between the calculation absorption spectra and experimental results. The experimental peak at 457 nm shows a 10 nm deviation from the averaged PE spectrum and a 40 nm deviation from the electrostatic spectrum. The larger deviation from the electrostatic model results further highlights the importance of accurate protein environment modeling. The comparison in Figure 4 emphasizes differences between the PE and electrostatic frameworks, underscoring the significance of polarizability and multipole effects on the absorption properties of the flavin chromophore originating from the protein environment.

Specifically, Figure 4B shows that the PE spectrum would require a smaller spectral shift of 35 nm to align with the 562 nm peak of an earlier experimental study,^9^ whereas the electrostatic model requires a spectral shift of 40 nm. It should be added here that while the electrostatic model is only marginally off on the first peak, the significant difference between PE and electrostatic models are the overall shape of the spectra, for which PE is much closer to the earlier experimental spectral profile.^9^ Additionally, peaks at shorter wavelengths align more closely with experimental findings compared to the electrostatic scenario. A previous theoretical study^12^ suggested two ultraviolet peaks at 330 and 340 nm, which are highlighted in Figure 4. Although the PE and electrostatic spectra appear similar in Figure 4, the PE spectrum shows two minor peaks at 330 and 340 nm. Moreover, no secondary peak is observed for the 330 nm peak for the electrostatic spectrum where in contrast the PE spectrum exhibits two peaks around 308 and 318 nm, indicating that the splitting of the peaks at 330 and 340 nm could stem from a lacking description of polarizable effects.

**Figure 5:**
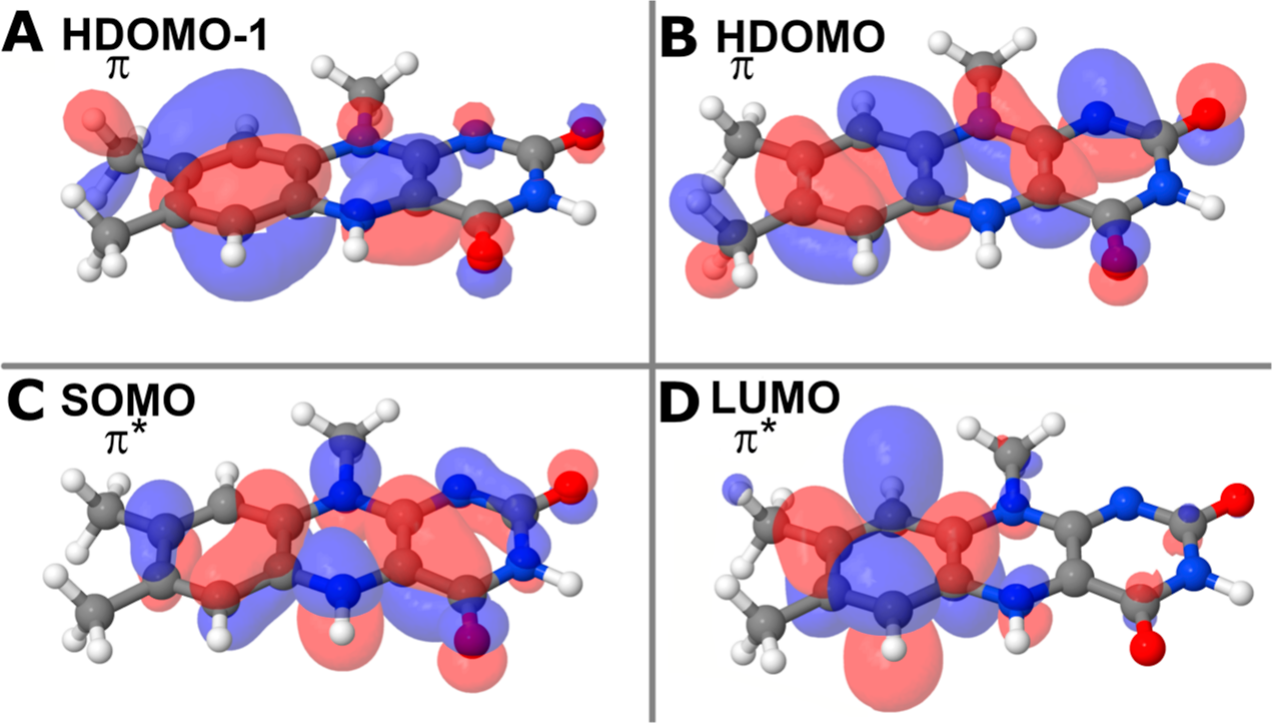
Prominent transition orbitals. The spectra in Figures 4 are composed of excitations between these orbitals. (A,B) Two highest doubly occupied molecular orbitals (HDOMO) are categorized as π orbitals. (C,D) Singly occupied molecular orbital (SOMO) and lowest unoccupied molecular orbital (LUMO) are π* orbitals. Orbitals are visualized with a cutoff value of 0.02 in Jmol.^70^

**Figure 6:**
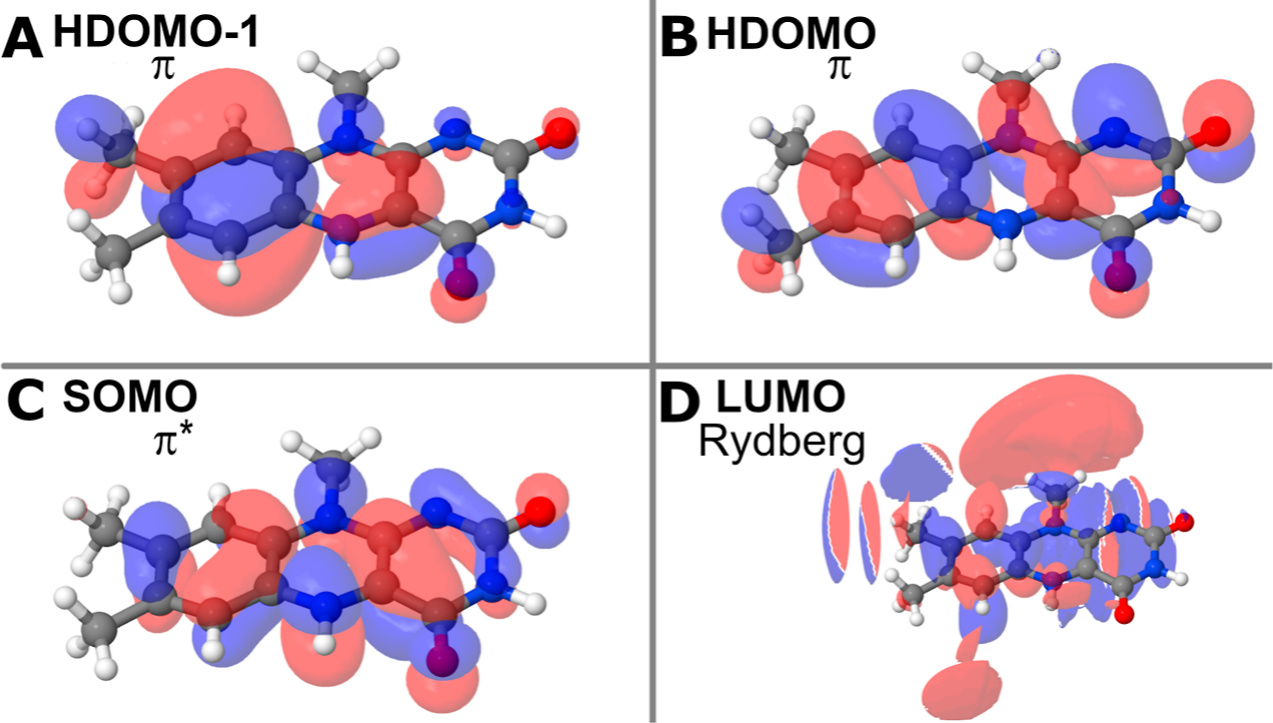
Orbitals of the most prominent transitions for the electrostatic calculations. (A,B) Two π orbitals with the red spheres representing the positive solution of the wave function with the negative solution being represented by the blue spheres. (C,D) Antibonding (π*) and a Rydberg orbital. The color scheme from (A) and (B) are preserved here. The orbitals have been illustrated using Jmol.^70^

Theoretical studies of photoexcitations often include a classification of electronic transitions.^11,71^ While molecular orbitals are no real observables, they still provide important information for understanding the electronic dynamics. The orbitals for the specific transitions indicated in Figure 3, are illustrated in Figure 5. A direct comparison of these transition orbitals with earlier investigations^11^ reveals inconsistencies, highlighting the potential impact of diverse environmental conditions that were included in the present study (see Figure 6). It should, however, be noted that previous studies on AtCry1 absorption that includes orbital analysis are a rather scarce amount.^9^

**Figure 7:**
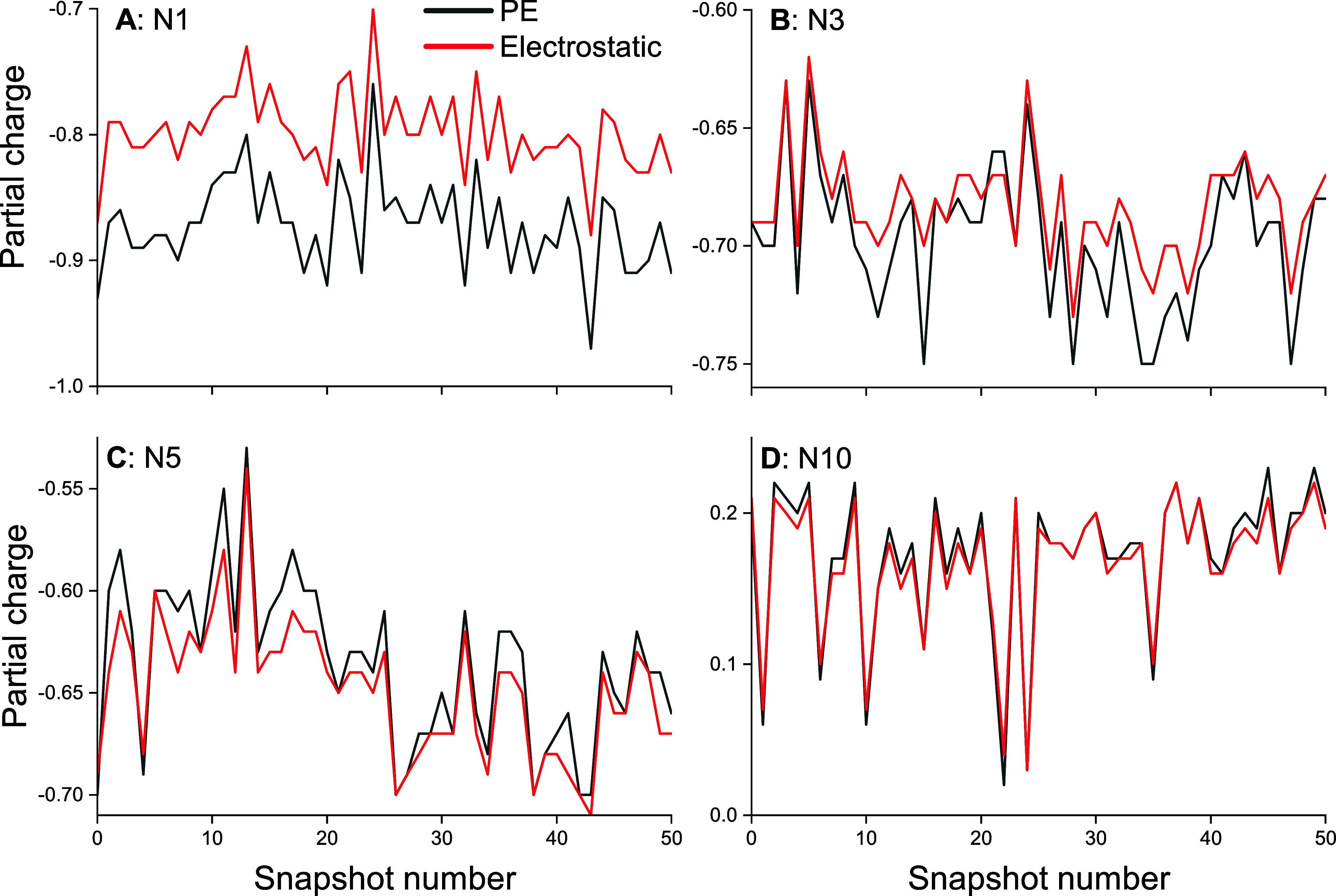
Mulliken charge analysis of the four nitrogen atoms of the flavin part of FADH^•^ with respect to the snapshot number, which is sampled every 10 ns of the MD trajectory.

Another aspect of investigating the influence of the environment on the flavin part of FADH^•^ is by calculating the partial charges of the Flavin. Figure 7 shows the partial charge difference of the four nitrogen atoms in both the PE and the electrostatic models. It is found that the inclusion of PE has a large impact on the N1 nitrogen (Figure 5A) where the difference between the two models is up to 0.1 elementary charge. It can be seen that due to the different configurations sampled by the MD trajectory, the partial charges fluctuate by up to about 0.2 elementary charge. Moreover, it is seen that the PE and electrostatic models are influenced similarly by the fluctuations of the environment, suggesting that influences of the environment are not dependent on the description level of the environment.

## Conclusions

In conclusion, we performed multiple TD-DFT calculations, approximating the protein environment as point charges with polarizabilities using the CP3 and SEP libraries. The resulting spectra were compared to earlier experimental spectra of the same system. From these comparisons, it could be concluded that the inclusion of PE in the calculations not only brought the spectra closer to the experimental right-most peak (see Figure 4) but also reduced the deviation of multiple peaks compared to electrostatic calculations, while at the same time maintaining an overall shape of the absorption spectrum, which was more similar to the experimental spectrum on multiple aspects compared to the electrostatic embedding. The results indicate that not only accounting for thermal effects but also polarizabilities is important when calculating absorption spectra theoretically. Our molecular orbital analysis reveals a significant change in the electronic structure between the two embedding approaches. The results suggest that the electronic structure of the excited states will be altered drastically by PE and that further investigations will be mandatory.
